# Composition and biodiversity of soil and root-associated microbiome in *Vitis vinifera* cultivar Lambrusco distinguish the microbial *terroir* of the Lambrusco DOC protected designation of origin area on a local scale

**DOI:** 10.3389/fmicb.2023.1108036

**Published:** 2023-02-22

**Authors:** Enrico Nanetti, Giorgia Palladino, Daniel Scicchitano, Giulia Trapella, Nicolò Cinti, Marco Fabbrini, Alice Cozzi, Giovanni Accetta, Carlo Tassini, Luigi Iannaccone, Marco Candela, Simone Rampelli

**Affiliations:** ^1^Unit of Microbiome Science and Biotechnology, Department of Pharmacy and Biotechnology (FaBiT), Alma Mater Studiorum—University of Bologna, Bologna, Italy; ^2^Fano Marine Center, The Inter-Institute Center for Research on Marine Biodiversity, Resources and Biotechnologies, Fano, Italy; ^3^Microbiomics Unit, Department of Medical and Surgical Sciences (DIMEC), University of Bologna, Bologna, Italy; ^4^Istituto Tecnico Statale “Ignazio Calvi”, Finale Emilia, Italy

**Keywords:** microbial terroir, microbiomes, *Vitis vinifera*, rhizosphere, plant growth-promoting bacteria

## Abstract

**Introduction:**

Wines produced from the same grape cultivars but in different locations possess distinctive qualities leading to different consumer’s appreciation, preferences, and thus purchase choices. Here, we explore the possible importance of microbiomes at the soil–plant interface as a determinant of the terroir properties in grapevine production, which confer specific growth performances and wine chemo-sensory properties at the local scale.

**Methods:**

In particular, we investigated the variation in microbial communities associated with the roots of *Vitis vinifera* cultivar Lambrusco, as well as with surrounding bulk soils, in different vineyards across the “Consorzio Tutela Lambrusco DOC” protected designation of origin area (PDO, Emilia Romagna, Italy), considering viticultural sites located both inside and outside the consortium in two different seasons (June and November 2021).

**Results:**

According to our findings, rhizospheric and soil microbiomes show significant structural differences in relation to the sampling site, regardless of seasonality, while endophytic microbiomes seem to be completely unaffected by such variables. Furthermore, a deeper insight into the microbial terroir of PDO areas highlighted the presence of some rhizospheric microorganisms enriched inside the consortium and characterizing the PDO regardless of both sampling season and farming strategy. These include *Bacillus*, *Paenibacillus*, and *Azospirillum*, which are all well-known plant growth-promoting bacteria.

**Discussion:**

Taken together, our results suggest a connection between soil and root microbiomes of *V. vinifera* cultivar Lambrusco and the local designation of origin, emphasizing the potential role of PDO-enriched plant growth-promoting bacteria in vine growing and final quality of the Lambrusco DOC wine.

## Introduction

Wine is a fermented product of paramount economic and cultural importance for the agri-food sector worldwide (Gamalero et al., 2020; Martinho and Domingues, 2021). Therefore, vineyards are widely distributed and *Vitis vinifera* is one of the most cultivated fruit crops around the globe (Pinto et al., 2014; Smyczek et al., 2020). The local-scale pedoclimatic variation, also known as *terroir*, is a matter of growing interest for wine production, because considered of vital importance for the determination of the local wine quality characteristics and consequent consumer’s appreciation, preferences, and purchase choices (Savelli et al., 2020; Spognardi et al., 2021). Indeed, wines produced from the same grape cultivars but belonging to different *terroirs* possess distinctive qualities and economic value. To legally protect such local regional products, many geographical pedigrees—such as the Protected Designation of Origin (PDO) in Europe—have been released. However, establishing which factors underlie connections between *terroir* properties and the specific wine-associated chemo-sensory properties remains difficult and is mainly ascribed to general environmental characteristics that affect grapevine growth and health (Leeuwen and Seguin, 2006).

Recent studies suggested that the specific microbial communities associated with *V. vinifera* may be a key element of the *terroir*, as microbiome processes essential for vine growing and wine production show spatially defined patterns linked to the vineyard location (Gilbert et al., 2014; Pinto et al., 2014; Bokulich et al., 2016; Novello et al., 2017). Vitulo et al. (2019), for instance, found that the geographic indication is a good driver of microbiome differentiation of the vine bark when comparing plants from two Italian wine-producing regions (Piedmont and Tuscany). Similar results were obtained by Mezzasalma et al. (2017), who defined a certain fraction of the grape berries microbiome that significantly varied in relation to the geographical area. The same association has been highlighted when investigating the soil and the root microbiomes, with distinct microbial characteristics for different viticultural regions that probably correspond to a regional-specific contribution to the qualities of the grapes and wine (Gilbert et al., 2014; Zarraonaindia et al., 2015; Coller et al., 2019; Aguilar et al., 2020; Biget et al., 2021; Gobbi et al., 2022).

In Italy, in pedoclimatic regions including well-defined delimitations of PDO production, the same grapes are cultivated inside and outside the PDO sites, with similar yields but different properties. This opens the question of the importance of microbiome variations at the soil–plant interface in determining the local *terroir* quality at the local scale, with the cascade implications for the PDO production. In order to provide some glimpses in this direction, we aim at investigating the presence of differences in the microbiome-dependent *terroir* features (rhizospheric, endophytic, and bulk soil microbiomes) in plant specimens of *V. vinifera* cultivar Lambrusco sampled across three different vineyards from the same pedoclimatic region but located inside and outside the “Consorzio Tutela Lambrusco DOC” PDO area, in Emilia Romagna, Italy. In particular, two vineyards were positioned immediately inside the PDO area, and another vineyard just outside the PDO area. In Emilia-Romagna, the Italian region leading Lambrusco’s production globally,^1^ it is of primary economic importance to safeguard the “Consorzio Tutela Lambrusco DOC” PDO. Indeed, with 42 million bottles sold in 2020,^2^ Lambrusco DOC is one of the best-selling Italian wines in the world. Moreover, its PDO territory overlaps with that of the Balsamic Vinegar of Modena (that is produced from the same grapes as Lambrusco DOC wine), which showed a production turnover of 370 million euros in 2021.^3^ For all these reasons, we think that a finer characterization of the microbial *terroir* on the boundaries of the PDO area can contribute to a better safeguard and enhancement of the production. Specifically, for capturing the full variation due to different agricultural practices, the two vineyards within the PDO area were subjected to different agronomic approaches, i.e., organic and conventional agriculture. Furthermore, to test the hypothesis that the composition and/or the diversity of the microbiome at the soil–root interface constitute a signature for defining and protecting a PDO area, we explored the microbiome structure at two time points (June and November) in order to get a full picture of the root-associated microbial *terroir* at different stages of plant maturation. All the plants included in this study were *V. vinifera* cultivar Lambrusco, grafted on the hybrid *Vitis berlandieri* × *Vitis riparia* KOBER 5BB. In addition to enriching our understanding of the importance of soil and root-associated microbiomes in defining the wine *terroir* and the relative PDO area, this study may provide further economic incentive for agricultural and oenological practices that safeguard regional microbial *terroir* and biodiversity.

## Materials and methods

### Study site

**Figure 1:**
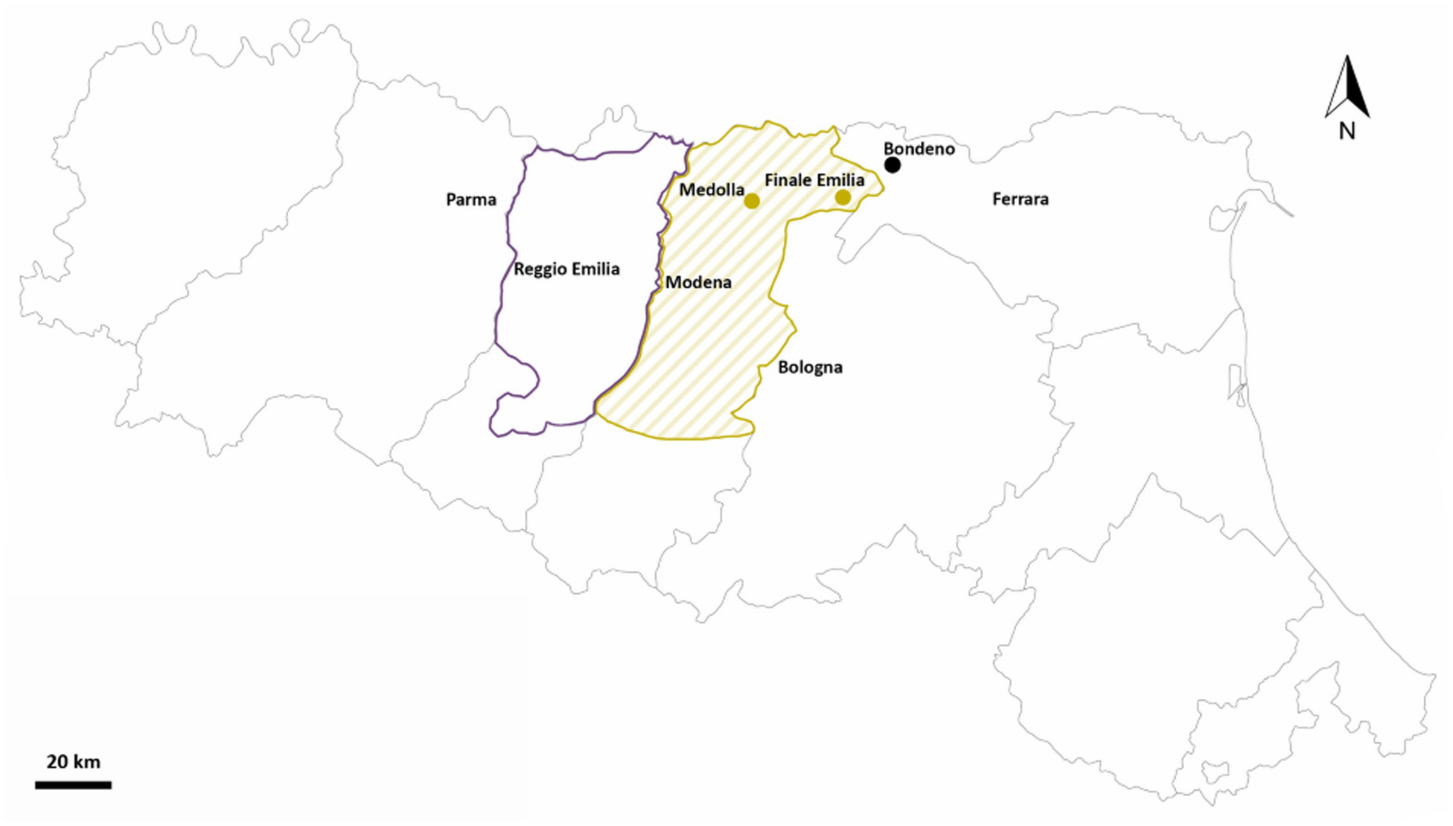
Sampling locations. Map of Emilia Romagna (Italy) showing the present study sampling sites in Bondeno (FE), Finale Emilia (MO) and Medolla (MO). Sampling locations are represented as yellow-green dots if located inside the Lambrusco DOC PDO viticultural area and as black dots if located outside the borders of such area. Borders of the entire “Consorzio Tutela Lambrusco DOC” are drawn (with a darker color for the Reggio Emilia territory and with a lighter color for the Modena territory).

Grapevine roots and soil samples were collected from three different vineyards in Emilia Romagna (Italy) across two timepoints. In particular, from each site, i.e., Bondeno (44.953 N/11.305 E, Ferrara), Finale Emilia (44.839 N/11.285 E, Modena), and Medolla (44.816 N/11.062 E, Modena; Figure 1), 15 plants and two bulk soils were retrieved both in June and November 2021 (immediately after the grape harvest), for a total of 90 root and 12 soil samples. Furthermore, for each root sample, the rhizospheric and endophytic microbial communities were both analyzed. The three vineyards considered in this study were characterized by different agronomic managements and biogeographical features. Specifically, both Finale Emilia and Medolla sites are located inside a protected designation of origin (PDO) area but differ in terms of the agricultural approach employed (chemical-based vs. organic, respectively) while the Bondeno site is found outside the PDO area and a traditional chemical-based farming approach is used. A schematic summary of samples distribution across the three sites and the two timepoints (June and November) is provided by Supplementary Table 1.

### Samples collection and pre-treatment

For the microbiome characterization, each plant root was investigated considering two different ecosystems, namely rhizospheric soil and root endophytic ecosystem, and samples of bulk soil were also analyzed, for a total of 102 samples (Supplementary Table 1). Grapevine thin lateral roots were collected after digging 10–20 cm under the plants. Bulk soil samples were collected near the area where plants were located, after removing the top centimeters of surface soil and the grass cover, if present. All samples were collected wearing sterile gloves, placed inside a sterile 50 ml Falcon tube and stored at −80°C until further processing.

In order to separate the two plant compartments (i.e., rhizosphere and endosphere), roots were thoroughly treated as previously described in D’Amico et al. (2018). In brief, approximately 3 cm of terminal roots portions, including tips, were dissected using sanitized scissors and tweezers to standardize the quantity of starting material. Then, root segments were placed in 15 ml Falcon tubes filled with 2.5 ml of modified PBS buffer (130 mM NaCl, 7 mM Na_2_HPO_4_, 3 mM NaH_2_PO_4_, pH 7.0, and 0.02% Silwet L-77) and left on a shaking platform at 180 rpm for 20 min to perform washing. After removing the roots, the washing buffer was centrifuged at 1.500 × *g* for 20 min and the resulting pellet was regarded as the rhizospheric soil. Roots were then re-washed under the same conditions and transferred to another 15 ml Falcon tube containing 2.5 ml of modified PBS buffer before undergoing 10 cycles of sonication as follows: 30-s pulses at 160 W with 30-s breaks in an ultrasonic bath (Branson 1800, Branson Ultrasonic Corporation, Danbury, CT, United States). After washing and sonication, roots were grinded by means of mortar and pestle in order to reach the root inner portions. This procedure led to a total of 180 samples (90 rhizospheres + 90 roots) that were analyzed together with the 12 bulk soil samples. All samples were kept frozen at −80°C until genomic DNA extraction.

### DNA extraction and sequencing

Total genomic DNA was extracted from all the 192 samples, i.e., bulk soils (0.25 g), rhizospheres (approximately 0.25 g), and smashed roots, using the DNeasy PowerSoil Kit (QIAGEN, Hilden, Germany) following the manufacturer’s instructions with minor modifications: a FastPrep instrument (MP Biomedicals, Irvine, CA, United States) was used for the homogenization step with a cycle consisting of three 1-min steps at 5.5 movements per sec with 5-min incubations in ice between each run and, at the end of the protocol, DNA elution was preceded by a 5-min incubation in ice. Then, DNA was quantified by using NanoDrop ND1000 (NanoDrop Technologies, Wilmington, DE) and diluted in PCR grade water to the final concentration of 5 ng/μl before amplification. Five microliters of diluted DNA were used as template for the PCR reaction. PCR was performed in a final volume of 50 μl containing 25 ng of genomic DNA, 2X KAPA HiFi HotStart ReadyMix (Roche, Basel, Switzerland) and 200 nmol/L of 341F and 785R primers carrying Illumina overhang adapter sequences for amplification of the V3–V4 hypervariable regions of the 16S rRNA gene. Specifically, the thermal cycle consisted of initial denaturation at 95°C for 3 min followed by 25 cycles of denaturation (95°C for 30 s), annealing (55°C for 30 s), and extension (72°C for 30 s), with a final extension step at 72°C for 5 min (Turroni et al., 2016). PCR amplicons were cleaned up with Agencourt AMPure XP magnetic beads (Beckman Coulter, Brea, CA, United States). Indexed libraries were prepared by limited-cycle PCR using Nextera technology. Indexing was followed by a second clean-up step, as already described, and then libraries were quantified using Qubit 3.0 fluorimeter (Invitrogen), normalized to 4 nM and pooled. Before sequencing, the sample pool was denatured with 0.2 N NaOH and diluted to 4.5 pM with a 20% PhiX control. Sequencing was performed on Illumina MiSeq platform using a 2 × 250 bp paired end protocol, according to the manufacturer’s instructions (Illumina, San Diego, CA, United States). Sequencing reads were deposited in the ENA archive with the accession code PRJEB57815.

### Bioinformatics and biostatistics

Raw sequences were analyzed using a pipeline which combines PANDAseq 2.11 (Masella et al., 2012) and QIIME2 2021.8.0 (Bolyen et al., 2019) for all 192 samples. High-quality reads (min/max length = 350/550 bp) were retained thanks to the “fastq filter” function of the Usearch 11.0.667 algorithm (Edgar, 2010) and then binned into amplicon sequence variants (ASVs) using DADA2 2021.8.0 (Callahan et al., 2016). Samples with less than 1,000 high-quality reads were discarded and not used for subsequent analyses. The VSEARCH algorithm 2021.8.0. (Rognes et al., 2016) and the SILVA database (December 2017 release; Quast et al., 2012) were employed for taxonomic classification. All unassigned and eukaryotic sequences were discarded. ASVs table was then rarefied to retain a number of 1,138 sequences per sample. The QIIME2 feature table rarefy plugin was used to perform rarefaction. Statistical analyses were carried out using the R software (R Core Team; www.r-project.org—last access: April 2022), v. 4.2.0, implemented with the packages “Made4” 1.72.0 (Culhane et al., 2005), “vegan” 2.6–4 (https://cran.r project.org/web/packages/vegan/index.html—last access: October 2022), “pairwiseAdonis” 0.4 (Martinez Arbizu, 2020), and STAT 0.1.0 (https://cran.r-project.org/web/packages/STAT/index.html—last access: April 2019). Beta diversity based on unweighted UniFrac distances was computed, and the separation of data in the Principal Coordinates Analysis (PCoA) was assessed with a permutation test with pseudo-F ratios (function “adonis” in the vegan package and function pairwiseAdonis in the homonymous package). Kruskal–Wallis test was used to assess significant differences in alpha diversity distributions between groups. Bacterial genera with the largest contribution to the ordination space were detected by the function envfit of the R package vegan on the genus relative abundances. *p* values, when necessary, were corrected for multiple testing by means of the Benjamini-Hochberg method, with a false discovery rate (FDR) ≤ 0.05 considered to be statistically significant. Linear discriminant analysis (LDA) effect size (LEfSe, Segata et al., 2011), aimed at identifying discriminant rhizospheric taxa between vineyards located inside and outside the Lambrusco DOC PDO viticultural area, regardless of the agricultural practices employed, was performed on genus-level relative abundance tables, retaining only taxa with LDA score threshold of ±2 (on a log10 scale) and value of *p* ≤ 0.2. The online Galaxy Version interface (https://huttenhower.sph.harvard.edu/galaxy/, last accessed in October 2022) was used to run LEfSe. All taxa identified by LEfSe, thus significantly enriched either inside or outside PDO sites, were then tested for their putative ability to support plant growth by the presence of some well-known plant growth-promoting (PGP) genes. For this purpose, starting from the QIIME2 genera level taxonomic assignment, an oligotyping procedure (Eren et al., 2014) was implemented to detect the species belonging to the genera previously identified by LEfSe through the “Minimum Entropy Decomposition” (MED) module and the global earth microbiomes (GEM) catalogue (November 2020 release; Nayfach et al., 2021). For each genus, the command line was “decompose <ASVs representative sequences fasta.file> − g-M 1-V5.” The-M integer defines the minimum substantive abundance of an oligotype, and the-V integer defines the maximum variation allowed in each node. The node representative sequence of each oligotype was used for species profiling with the QIIME2 feature-classifier plug-in (Bolyen et al., 2019), selecting the VSEARCH algorithm 2021.8.0 (Rognes et al., 2016) and the GEM database (Nayfach et al., 2021). Then, the aminoacidic sequence of some well-characterized PGP proteins, obtained from the reference sequence of the NCBI protein database (https://www.ncbi.nlm.nih.gov; accessed from the 1st to the 31st October 2022), was recovered and blasted against the non-redundant protein sequences NCBI database selecting as target organisms for our queries the bacterial taxa identified by the oligotyping procedure. Unclassified members of a specific taxon were considered when it was impossible to assign the ASVs at the species level, or when the number of oligotypes not assigned at the species level but assigned at higher taxonomic levels overcame the number of species-level matches. Alignments were filtered according to a query coverage of at least 40% and an alignment percentage of identity of at least 20%. The PGP functions selected for our analysis were: nitrogen fixation, phosphorous solubilization, iron chelation, production of the phytohormone indole-3-acetic acid (IAA), and production of the enzyme 1-aminocyclopropane-1-carboxylic acid (ACC) deaminase. For each of these functions, we selected some marker genes from previous scientific literature resulting in 15 protein sequences recovered and blasted. Specifically, the chosen marker genes were NifB, NifE, NifH, NifN, NifV, and NifU (i.e., nitrogen fixation genes) for nitrogen fixation, the alkaline phosphatase phoA, and the glucose dehydrogenase GDH for phosphorous solubilization, three markers of two relevant bacterial siderophores for iron chelation (namely EntF/EntS for enterobactin and FslA for rhizoferrin), three genes directly involved in IAA synthesis (i.e., ipdC, aro10, and aldH) and the AcdS gene encoding the enzyme ACC deaminase (see Supplementary Table 3 for genes accession and version numbers). Moreover, the presence of the same marker genes was verified across the entire rhizospheric microbiome by means of Phylogenetic Investigation of Communities by Reconstruction of Unobserved States (PICRUSt2 v. 2.5.0) analysis (Douglas et al., 2020). Notably, during the process of ASVs sequences matching to the KEGG database (Wixon and Kell, 2000, queried on January 10, 2023), two out of 15 reference proteins (i.e., FslA and aro10) were not found in the KEGG database (Supplementary Table 4).

Bacterial co-abundance groups (CAGs) were determined as formerly described by Schnorr et al. (2014). In brief, the Kendall correlation test was used to evaluate the associations among bacterial genera, which were visualized using hierarchical Ward clustering with a Spearman correlation distance metric and used to define CAGs at the genus level. The significant associations observed were controlled for multiple testing with the *q* value method (FDR ≤ 0.05; Dabney et al., 2013). Permutational multivariate ANOVA (PERMANOVA; Anderson, 2001) was employed to verify whether the CAGs were significantly different from one another. The Wiggum plot network analysis was carried out using cytoscape software v. 3.9.1 (http://www.cytoscape.org/, last accessed in November 2022) as previously described (Claesson et al., 2012).

## Results

### Microbiome composition and biodiversity in soil, rhizosphere, and root samples from viticultural farms located inside and outside the Lambrusco DOC PDO viticultural area

A total of 90 *V. vinifera* Cultivar Lambrusco roots samples and 12 bulk soil samples were taken from three different viticultural farms in June and November 2021 in Emilia Romagna, Italy (Figure 1). In particular, from each vineyard (located in Bondeno, Finale Emilia and Medolla) 30 roots (15 in June and 15 in November) and four bulk soils (two in June and two in November) were retrieved, resulting in 102 samples. Among those, all the 90 roots were treated as previously described in order to separate the rhizospheric from the endophytic compartment, leading to a total of 180 *V. vinifera* samples and 12 bulk soil samples. The selected farms were characterized by different designation of origin and by different agricultural practices: (i) Bondeno (non-PDO area, conventional farming), (ii) Finale Emilia (PDO area, conventional farming), and (iii) Medolla (PDO area, organic farming). For the three sites and the two timepoints, microbiome compositional structure was investigated by NGS sequencing of the 16S rRNA gene (V3–V4 hypervariable regions), resulting in ≃1,5 M high-quality reads, with an average of 9,581 ± 2,329 reads per sample (mean ± SD), which were binned in 31,264 ASVs (samples with less than 1,000 high-quality reads were not analyzed).

**Figure 2:**
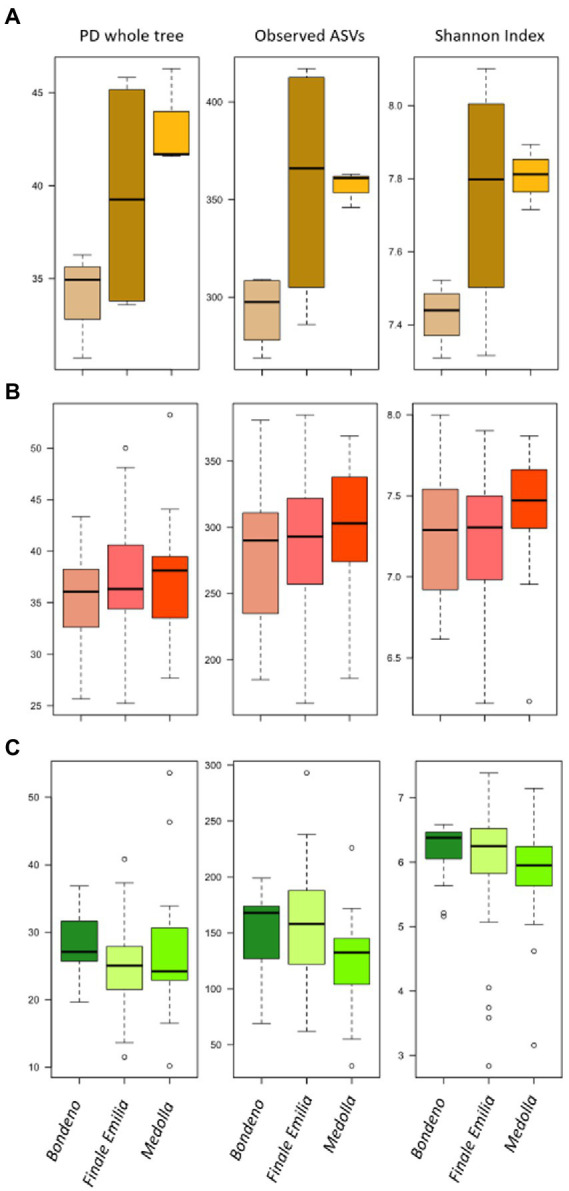
Alpha diversity of bulk soil and of *Vitis vinifera* rhizosphere and root microbiomes in the three studied farms. Box-plots showing the distributions of the Faith’s Phylogenetic Diversity (PD whole tree), Observed ASVs and Shannon Index calculated for the bulk soil **(A)**, the rhizosphere **(B)**, and the root **(C)** in the three sampled vineyards (located in Bondeno, Finale Emilia, and Medolla). The only significant differences were observed for the bulk soil samples (*p* ≤ 0.05, Kruskal–Wallis test).

Firstly, the bulk soil microbiome was characterized by a significantly higher degree of biodiversity with respect to both rhizospheric and endophytic compartments (*p* ≤ 0.05, Kruskal–Wallis test, Supplementary Figure 1). When we sought for differences among farms, we only observed a gradual increase of the soil biodiversity from Bondeno, to Finale Emilia and Medolla, with a trajectory that mirrored the path from non-PDO to PDO area and from conventional to organic management. However, these differences are only appreciable at soil level, with the rhizosphere and root compartments from the different farms showing comparable levels of biodiversity (Figure 2).

**Figure 3:**
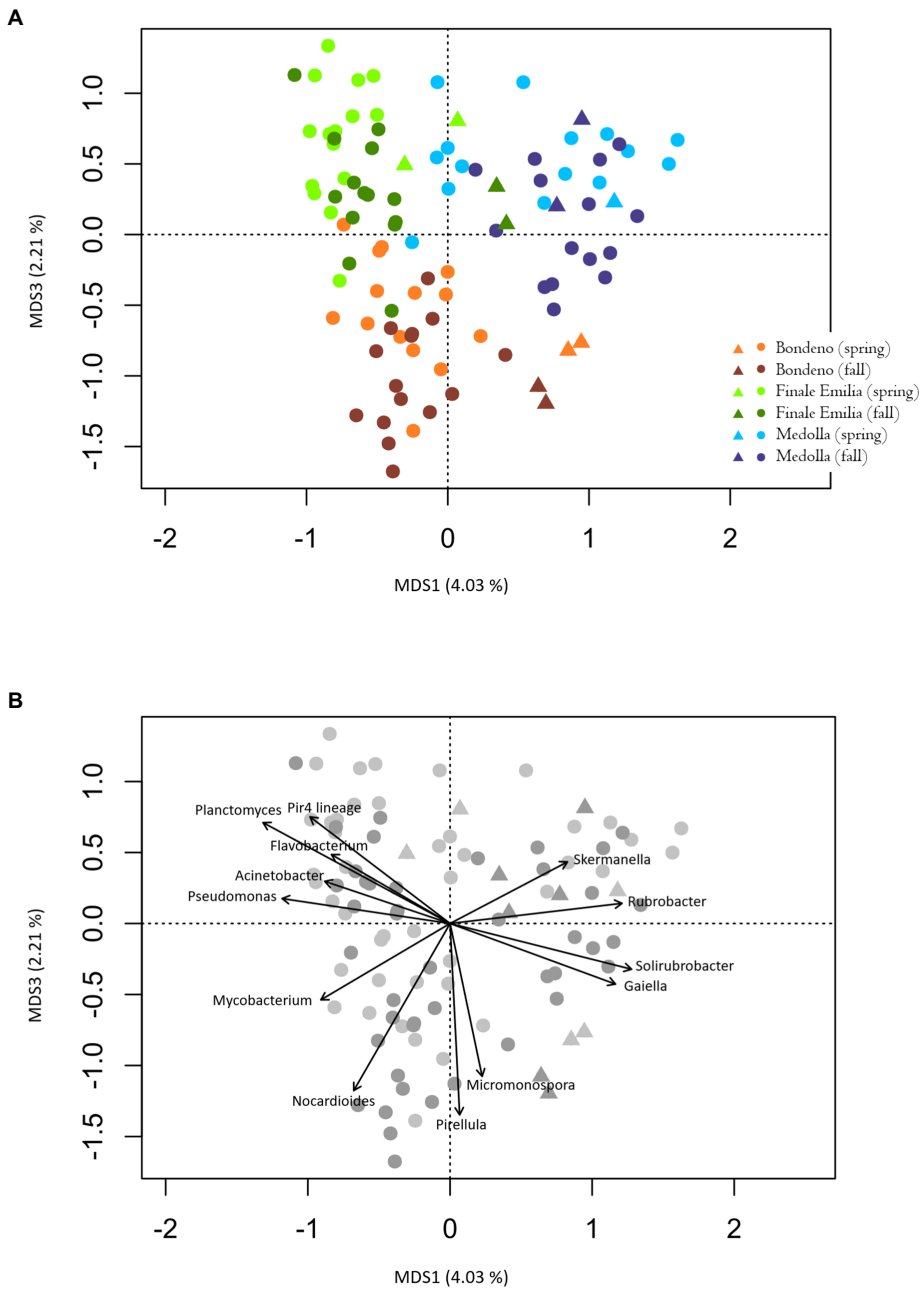
**(A)** Principal Coordinates Analysis (PCoA) based on unweighted UniFrac distances showing the variation of *Vitis vinifera* rhizosphere (dots) and bulk soil (triangles) microbiomes across sites, i.e., Bondeno (orange-red), Finale Emilia (green), and Medolla (blue) and seasons (lighter shades for spring and darker shades for fall; permutation test with pseudo *F*-ratio, *p* ≤ 0.001). The first and third principal components (MDS1 and MDS3) are plotted and the percentage of variance in the dataset explained by each axis is highlighted. **(B)** The same graph as in **(A)** has been reprinted in order to visualize the bacterial genera most contributing to segregations, whose relative abundance was superimposed in the PCoA plot (function envfit of the R package vegan) considering only genera with a *p* ≤ 0.001.

Beta-diversity analysis revealed a clear pattern toward segregation of the rhizospheric microbial communities according to the sampling location, but not to the sampling season, as shown by the unweighted UniFrac distances (permutation test with pseudo *F*-ratio, *p* ≤ 0.001; Figure 3A). Interestingly, the same PCoA indicates that a similar trend can be observed also for the bulk soils, as if the differences detected into the rhizosphere compartment mirrored differences in the soil. In order to identify those bacterial genera most contributing to the separation of the rhizospheric samples in the PCoA, the relative abundances of such taxa were superimposed in the unweighted UniFrac beta diversity plot (Figure 3B). Our results indicate that some bacterial genera are more represented in a particular farm regardless of the season. Specifically, the genus *Pirellula*, *Micromonospora*, and *Nocardioides* are the most characteristic of the Bondeno farm, while *Pseudomonas*, *Flavobacterium*, *Acinetobacter*, *Pir4 lineage*, and *Planctomyces* can be associated with Finale Emilia samples and, finally, *Skermanella*, *Gaiella*, *Solirubrobacter*, and *Rubrobacter* are the most distinguishing of the Medolla farm. Conversely, it is noteworthy to point out that the only ASVs detected across the entire rhizospheric cohort were assigned to uncultured members of the *Planctomycetaceae* and to uncultured members of the *Tepidisphaeraceae*. For these taxa, coefficients of variations were 0.4 (mean ± SD % rel. ab., 3.6 ± 1.5) and 0.6 (mean ± SD % rel. ab., 1.7 ± 1.0), respectively, meaning that these taxa were present in the rhizospheres at comparable levels, independently of site and season, constituting a sort of core bacterial group for *V. vinifera* cultivar Lambrusco. Interestingly, the root samples show no significant structural differences across different sites and seasons (permutation test with pseudo *F*-ratio, *p* > 0.05) and a sharp segregation appears in the PCoA only when comparing the endophytic cluster with the entire set of the bulk soil samples (permutation test with pseudo *F*-ratio, *p* ≤ 0.001; Supplementary Figure 2).

**Figure 4:**
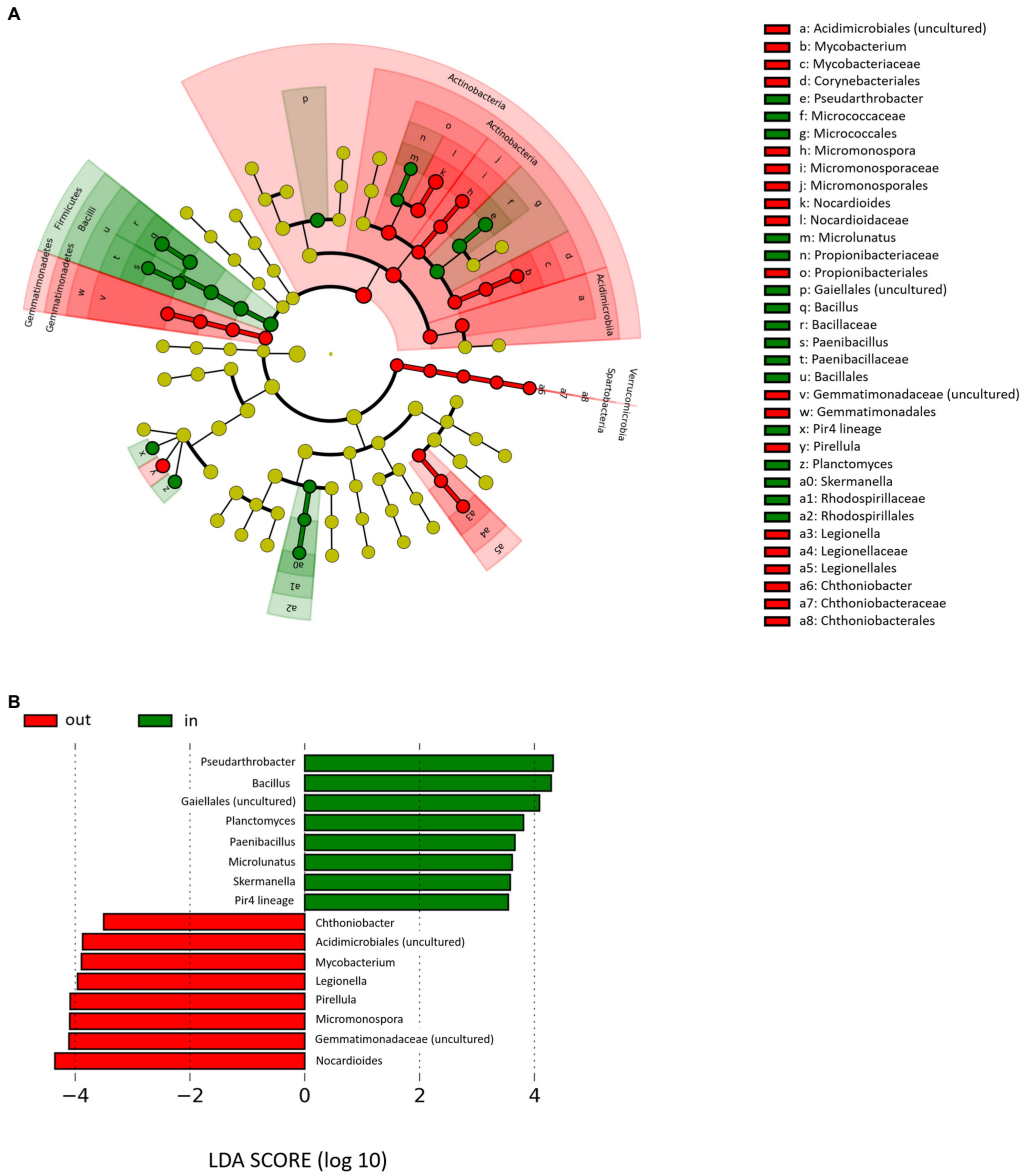
Rhizospheric microbiome signatures of PDO production sites. **(A)** Cladogram of microbial taxa differentially represented between farms located inside (Finale Emilia and Medolla, green) and outside (Bondeno, red) the PDO area at phylum to genus level. Only genera whose relative abundance was higher than 0.5% in at least 33% of the rhizospheric samples are represented. The diameter of each circle is proportional to the genera relative abundance within the entire rhizospheric cohort. **(B)** Linear discriminant analysis (LDA) scores of discriminating genera between the abovementioned groups (the logarithmic threshold for discriminative features was set to 2.0). Plots were obtained by LDA effect size (LEfSe) analysis.

The LEfSe was finally used to identify rhizospheric bacterial genera that discriminated PDO-associated from non-PDO microbiomes, regardless of farming site, season and type of management (Figure 4). In particular, genera associated with Lambrusco DOC PDO area were *Bacillus*, *Pseudarthrobacter*, unclassified members of the order Gaiellales, *Planctomyces*, *Skermanella*, *Pir4 lineage*, *Microlunatus*, and *Paenibacillus*. On the other hand, genera less representing the PDO area were *Nocardioides*, *Micromonospora*, and *Pirellula*, unclassified members of the family *Gemmatimonadaceae* and of the order Acidimicrobiales, *Mycobacterium*, *Legionella*, and *Chthoniobacter*. Notably, such taxa were also generally more represented into the correspondent bulk soil microbiome, with the exception of *Pseudarthrobacter* and *Pir4 lineage* for what concerns the PDO area and *Nocardioides*, *Legionella*, and *Chthoniobacter* for what concerns the non-PDO area (Supplementary Table 2).

### Seasonal variations of the *Vitis vinifera* rhizospheric microbiome network across the Lambrusco DOC PDO region

**Figure 5:**
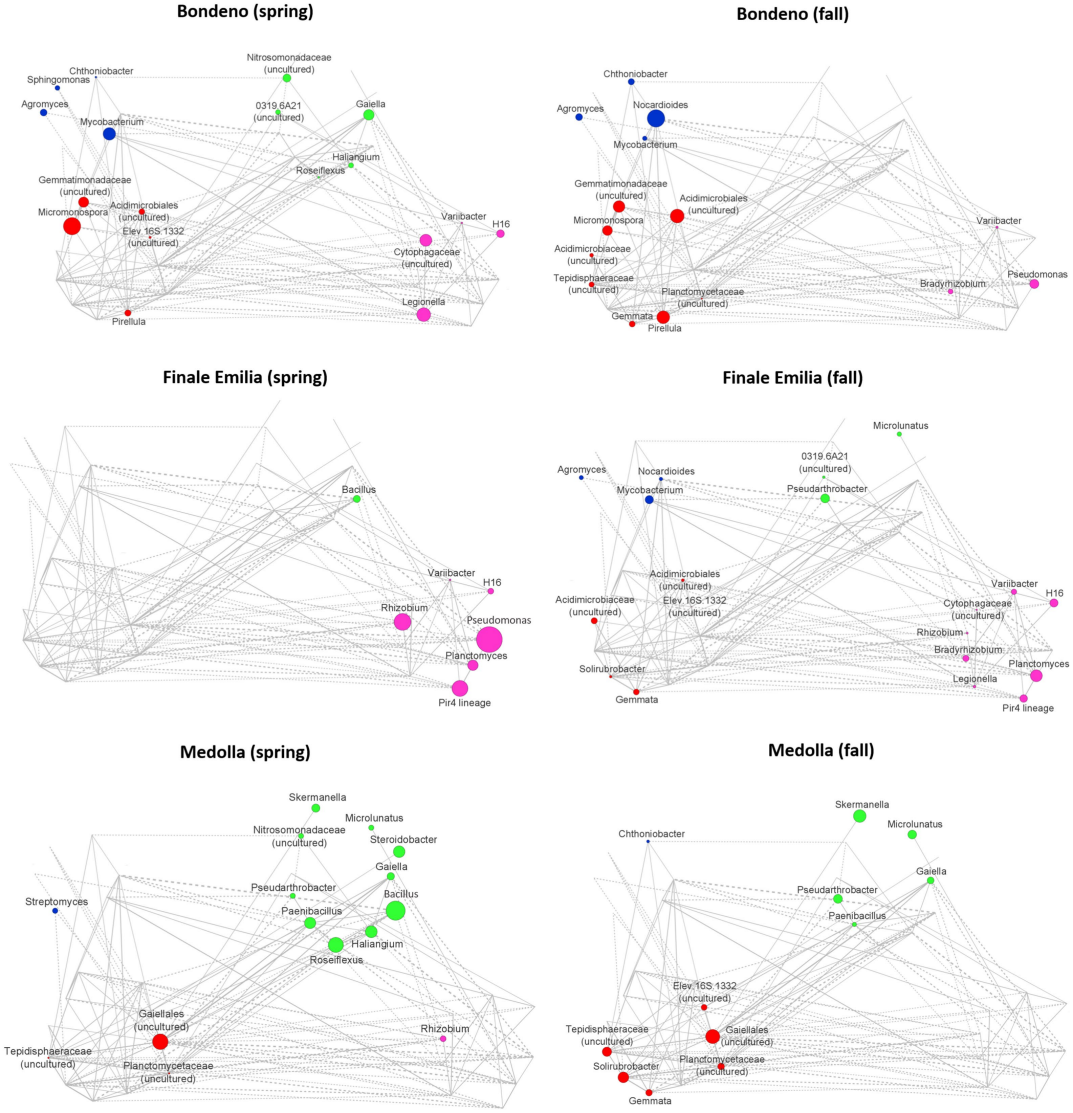
Declination of rhizospheric *Vitis vinifera* co-abundance groups according to the sampling site and to seasonality. Co-abundance groups (CAGs) are named according to the dominant bacterial genus in each group: *Pirellula* (red), *Nocardioides* (blue), *Pseudomonas* (pink), and *Bacillus* (green). Each node represents a bacterial genus and the size of the corresponding circle is proportional to its over-abundance on the average value within the population. The connections between nodes constitute positive (solid lines) and negative (dashed lines) Kendall correlations between genera (FDR ≤ 0.05). For CAGs definition see Supplementary Figure S3.

To identify specificities of the rhizospheric microbiome structure associated with the Lambrusco DOC PDO region and seasonality, we established co-abundance associations of genera and then clustered correlated bacterial taxa into four co-abundance groups (CAGs), describing microbiome configurations across the entire dataset (Supplementary Figure 3). The dominant (i.e., the most abundant) genera in these CAGs were *Pirellula* (red), *Nocardioides* (blue), *Pseudomonas* (pink), and *Bacillus* (green). The network-establishing CAGs relationships are named Wiggum plots, where genera abundances are represented as a circle proportional to the genus normalized over-abundance (Figure 5). The microbiome variation from Bondeno to Finale Emilia and Medolla through the two different seasons was accompanied by distinctive CAGs dominance, and most relevantly by abundances of the *Pirellula* and *Nocardioides* CAGs (Bondeno), the *Pseudomonas* CAG (Finale Emilia) and the *Bacillus* CAG (Medolla). When we sought for shared network topological features among Finale Emilia and Medolla microbiome structure, distinctive of the PDO region and not included in the control site (Bondeno), we found that nodes corresponding to *Bacillus* and *Rhizobium* were over-abundant during spring, whereas *Pseudarthrobacter* and *Microlunatus* nodes were over-abundant in the fall season. When combined, such results underline a sort of seasonal dynamic, very peculiar to the Lambrusco DOC PDO region independently of the type of management. Remarkably, most of these taxa constitute a subgroup of the species previously identified by LEfSe.

### Understanding the importance of PDO-related taxa for grapevine biology

**Figure 6:**
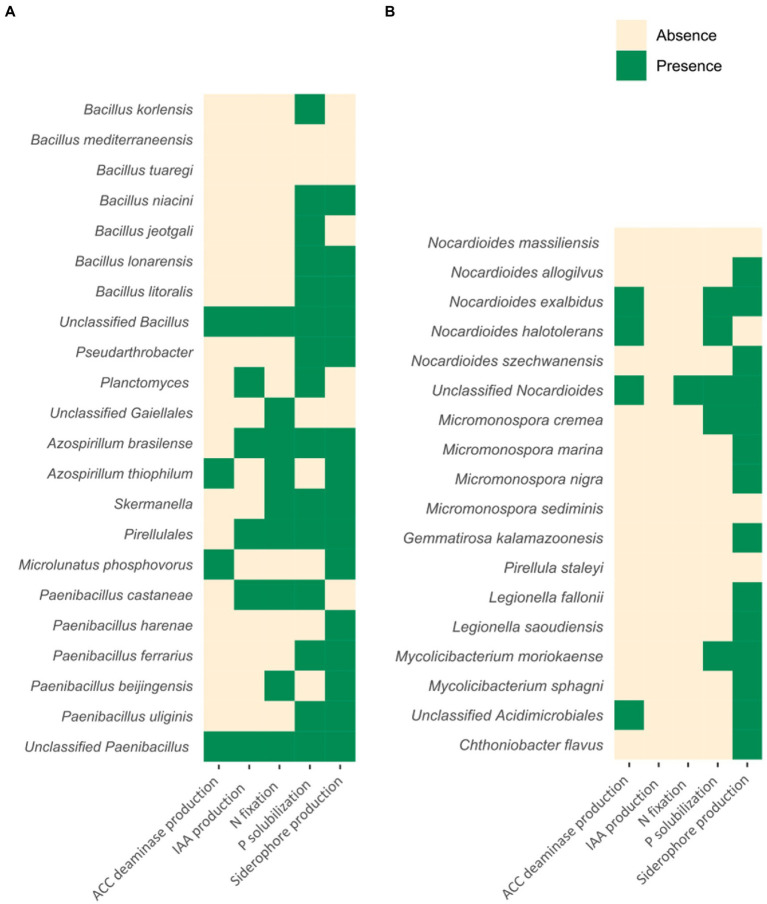
Schematic illustration showing the potential presence of PGP traits within the genomes of PDO-related **(A)** and non-PDO related **(B)** rhizospheric species. Each taxon was tested for the presence/absence of a specific set of PGP functions. The selected functions were nitrogen fixation, phosphorous solubilization, iron chelation, production of indole-3-acetic acid (IAA), and production of 1-aminocyclopropane-1-carboxylic acid (ACC) deaminase. Green squares for putative presence of PGP activities, ivory squares for absence of PGP activity.

Plant growth-promoting microorganisms regulate plant physiological reactions and foster plant growth with several mechanisms. Here, we sought for some of these functions within the reference genomes of the taxa revealed by LEfSe. Specifically, we first used oligotyping (Eren et al., 2014) to identify the bacterial species (or higher taxonomic levels in some cases, as explained above) nested by the ASVs sequences belonging to the genera identified by LEfSe. In particular, ASVs sequences coding for *Bacillus*, *Pseudarthrobacter*, *Planctomyces*, *Paenibacillus*, *Microlunatus*, *Skermanella*, *Pir4 lineage*, and uncultured members of Gaiellales (i.e., the PDO-related taxa identified by LEfSe) along with ASVs sequences coding for *Chthoniobacter*, *Nocardioides*, *Micromonospora*, *Pirellula*, *Legionella*, *Mycobacterium*, and uncultured members of Acidimicrobiales and *Gemmatimonadaceae* (i.e., the non-PDO taxa identified by LEfSe) were processed using the “Minimum Entropy Decomposition” (MED) module and the global earth microbiomes (GEM) catalogue (Nayfach et al., 2021). We found that *Bacillus korlensis*, *Bacillus mediterraneensis*, *Bacillus tuaregi*, *Bacillus niacini*, *Bacillus jeotgali*, *Bacillus lonarensis*, and *Bacillus litoralis*, unclassified species of the genus *Bacillus*, species of the genus *Pseudarthrobacter*, species of the genus *Planctomyces*, unclassified species of the order Gaiellales, *Azospirillum brasilense*, *Azospirillum thiophilum*, species of the Pirellulales order, *Microlunatus phosphovorus*, *Paenibacillus castaneae*, *Paenibacillus harenae*, *Paenibacillus ferrarius*, *Paenibacillus beijingensis*, and *Paenibacillus uliginis*, and unclassified species of the genus *Paenibacillus*, were the taxa characterizing the PDO-area. Notably, when applying oligotyping and GEM database, the ASVs sequences previously assigned to *Skermanella* were assigned to *A. brasilense* and *A. thiophilum*. We chose to retain both *Skermanella* and *Azospirillum* genomes for the following analysis, also because of the high level of overlapping found between the 16S rRNA sequences of these two taxa (Zhu et al., 2014). On the other hand, *Nocardioides massiliensis*, *Nocardioides allogilvus*, *Nocardioides exalbidus*, *Nocardioides halotolerans*, and *Nocardioides szechwanensis*, unclassified species of the genus *Nocardioides*, *Micromonospora cremea*, *Micromonospora marina*, *Micromonospora nigra*, *Micromonospora sediminis*, *Gemmatirosa kalamazoonesis*, *Pirellula staleyi*, *Legionella fallonii*, *Legionella saoudiensis*, *Mycolicibacterium moriokaense*, and *Mycolicibacterium sphagni*, unclassified species of the order Acidimicrobiales and *Chthoniobacter flavus* were the taxa most distinguishing the non-PDO area. Interestingly, the oligotyping procedure and the GEM database identified *Mycolicibacterium* species nested in the ASVs belonging to the genus *Mycobacterium*. In this regard, a recent comprehensive phylogenomic study by Gupta et al. (2018) revealed that *Mycolicibacterium* can be actually regarded as a distinct clade previously classified as *Mycobacterium* and now forming a novel microbial genus. Then, in order to investigate the presence of potential PGP traits related to all these microorganisms, the NCBI reference genomes of all of these taxa were scanned for genes associated with nitrogen fixation (essential for plant growth), phosphorous solubilization (important for plant P uptake), siderophore production (for growth in iron-limiting conditions), indole-3-acetic acid (IAA) phytohormonal secretion (beneficial to increase water and nutrient absorption), and 1-aminocyclopropane-1-carboxylic acid (ACC) deaminase (for ethylene precursor degradation and regulation of plant stress response). Among the taxa identified by LEfSe as PDO-characterizing, species belonging to the genera *Bacillus*, *Skermanella*/*Azospirillum*, *Paenibacillus*, and unclassified species of the order Pirellulales contained most of the PGP features, whereas species from the genera *Pseudarthrobacter*, *Planctomyces*, and *Microlunatus*, together with unclassified members of the order Gaiellales, contained only one or two out of the five investigated PGP traits (Figure 6A). Conversely, if we look at the microbial taxa related to the non-PDO area (Figure 6B), species of the genus *Nocardioides* (i.e., unclassified *Nocardioides* and *N. exalbidus*) are the only ones in which more than two PGP traits out of five have been detected. Furthermore, two important PGP functions, namely nitrogen fixation and IAA production, have been scarcely observed in non-PDO related taxa (with the first only detected in species of unclassified *Nocardioides* while the latter entirely absent in non-PDO related taxa). PICRUSt2 confirmed most of the findings (Douglas et al., 2020), with some exceptions, above all for what concerns siderophore production and ACC deaminase production (Supplementary Table 5). This can be attributed at least in part to the fact that two markers used in our analysis and necessary for predicting the functionalities are absent in the databases provided with PICRUSTt2.

## Discussion

This study aimed at characterizing the soil–plant microbiome dimension of the viticultural *terroir* of *V. vinifera* cultivar Lambrusco from the “Consorzio Tutela Lambrusco DOC” PDO area, in Emilia Romagna, Italy. This was made possible by comparing microbiomes from viticultural farms—of the same age—located immediately inside or outside the PDO area, thus controlling for complex variations associated with differences in pedoclimatic regions. The amplicon sequencing revealed a clear differentiation of the soil and rhizospheric microbiomes according to the sampling location, but not to the sampling season, and the bulk soil microbial diversity was higher within the PDO area rather than outside. When we applied the LEfSe analysis, we detected eight bacterial genera significantly differentially recruited by the plants grown inside the PDO consortium with respect to the non-PDO area. Specifically, the eight genera, and where possible the nested species, included *Bacillus* (*B. korlensis*, *B. mediterraneensis*, *B. tuaregi*, *B. niacini*, *B. jeotgali*, *B. lonarensis*, *B. litoralis*, and other unclassified species), *Pseudarthrobacter*, *Planctomyces*, *Paenibacillus* (*P. castaneae*, *P. harenae*, *P. ferrarius*, *P. beijingensis*, *P. uliginis*, and other unclassified species), *Microlunatus* (*M. phosphovorus*), and *Skermanella/Azospirillum* (*A. brasilense* and *A. thiophilum*), *Pir4 lineage* and uncultured members of the order Gaiellales. Stinkingly, such group of bacteria was also detected in the correspondent bulk soil samples of the same PDO areas, emphasizing the commonly accepted hypothesis that soil can function as rich microbial *reservoir* for those microorganisms that interact with the plant holobiont at the root level (Singh et al., 2019; Ling et al., 2022). Even if not entailing significant variations in the microbiomes compositional structure, seasonality was shown to be associated with relevant changes in the rhizosphere microbiome network topology, with features characterizing the seasonal dynamics in the PDO area. Interestingly, most of the network seasonal variations related to the PDO area involved PDO-related taxa, which seem to modulate their abundance in response to seasonality. All the PDO-related species identified by LEfSe (except for *B. mediterraneensis* and *B. tuaregi*) presented at least one of the PGP traits potentially involved in the biostimulation and biofertilization of grapevine, with some species combining multiple PGP traits, such as *Bacillus*, *Skermanella*/*Azospirillum*, and *Paenibacillus*, possibly exerting a multifactorial probiotic role for the plant growth and biology. In particular, the PGP features detected in PDO-related species included the abilities to produce ACC deaminase, IAA, and siderophores, of solubilizing phosphorous from soil particles and soil organic matter and of biofertilizing soil through nitrogen fixation, which are all features that play an important role in microbiome-root crosstalk and plant growth/adaptation (Qu et al., 2020). With such a specific microbiome configuration, PDO-related bacteria may induce modification of the root system architecture as previously demonstrated (Baldan et al., 2015), and thus enhance nutrients and water uptake by the grapevines, with a resulting higher resistance to environmental stresses, better plant health and, consequently, improvement of the organoleptic properties of the Lambrusco wine, probably contributing to the regional *terroir* (Rolli et al., 2015, 2017; Zarraonaindia et al., 2015). Conversely, non-PDO-related species show far less PGP traits. In this regard, the only widespread function identified is connected to the production of some well-known siderophores.

Referring to the available literature on the PDO-related bacteria, we noticed that *Bacillus* is widely found on the root of grapevines in several different studies (Longa et al., 2017; D’Amico et al., 2018; Marasco et al., 2018; Berlanas et al., 2019). In particular, the higher abundance of *Bacillus* in the PDO area is quite interesting since *Bacillus* is a well-known plant growth-promoting rhizobacterium which can have many beneficial effects on plant growth (Hashem et al., 2019). These include, for instance: improvement of iron acquisition (Xu et al., 2019), regulation of the Na+/K+ efflux (Tahir et al., 2019), and modulation of plant physiology by IAA production (Ansari et al., 2019). Additionally, *Bacillus* is able to promote plant root length, photosynthetic pigment formation, and shoot germination through the production of the ACC deaminase enzyme, which also enhances tolerance to salinity stress (Din et al., 2019). Specifically, for grapevine plantlets, it has been shown that *Bacillus* can upregulate melatonin synthesis and reduce the production of malondialdehyde and reactive oxygen species in salt and drought stress conditions (Jiao et al., 2016). Further, when we sought for plant growth-promoting features of *Paenibacillus*, we found that it can be important for enhancing drought tolerance by upregulating dehydration-responsive genes, RD29A and RD29B (Liu et al., 2020), and for improving root surface area, root projection area and root fork numbers by IAA production, nitrogen fixation, and phosphorous solubilization (Navarro-Noya et al., 2012; Li et al., 2022). Functional genes related to plant growth-promoting activity were also previously identified in *Pseudarthrobacter*, that is an aerobic auxin-producing bacterium (Park et al., 2020), *Azospirillum*, a noteworthy diazotrophic microorganism which stimulates plant growth in different ways, e.g., by enhancing roots development and lateral root formation by IAA production (Arzanesh et al., 2011; Fukami et al., 2018) and *M. phosphovorus* that has been reported as phosphorous accumulator in wastewater treatment plants (Dorofeev et al., 2020).

Finally, our data clearly show that all the above-mentioned PDO microbiome specificities are limited to the soil and rhizospheric microbiome ecosystems, while the corresponding root microbiomes, possibly under a strong host-driven selection pressure (Liu et al., 2017; D’Amico et al., 2018; Afzal et al., 2019), remain constant in the three different farms, independently of the PDO or non-DOP location.

Collectively, all the taxa we found characterizing the PDO area are commonly detected in grapevine rhizospheres (Zarraonaindia et al., 2015; Longa et al., 2017; Novello et al., 2017; D’Amico et al., 2018; Marasco et al., 2018; Berlanas et al., 2019; Bettenfeld et al., 2021). However, here their concomitant presence at high abundance, their network structure and their characteristic seasonal dynamics may represent a key feature of the “Consorzio Tutela Lambrusco DOC” microbial *terroir*, possibly contributing to the peculiarity of the regional wine product, generally supporting the strategic importance of the soil–plant microbiome interface in defining microbiome-associated *terroir* specificities of relevance for the overall product quality. Future studies on higher number of sites within and outside the PDO area, based on shotgun metagenomics and possibly providing for a more extensive sampling, are needed to better unravel the contribution of the root-associated microbiomes, as well as of specific PGP species and/or strains, to the specific regional characteristics of grapevines and associated local products. Finally, examining in depth the link between root microbiome and grapevine may also provide helpful information for vineyard management, productivity and precision oenology, as well as elements to be safeguarded as pivotal features of the microbial *terroir* of Lambrusco grapevine, especially in the context of the current global change scenario, where we are witnessing a continuous loss of microbial diversity in several ecosystems, including soil.

## Data availability statement

The data presented in the study are deposited in the ENA archive (https://www.ebi.ac.uk/ena/), accession number PRJEB57815.

## Author contributions

MC and SR designed research project and acquired funds. SR, GA, CT, and LI performed sampling activity. EN, DS, GP, and GT performed laboratory experiments. MC contributed to analytical tools and laboratory equipment. EN, SR, AC, MF, and DS analyzed the data. EN and SR wrote the original draft. GP, DS, GT, NC, MF, AC, GA, CT, LI, and MC reviewed and edited the paper. All authors contributed to the article and approved the submitted version.

## Funding

This work was supported by the “Controlling Microbiomes Circulations for Better Food Systems” (CIRCLES) project, which was funded by the European Union’s Horizon 2020 research and innovation program under grant agreement no. 818290.

## Conflict of interest

The authors declare that the research was conducted in the absence of any commercial or financial relationships that could be construed as a potential conflict of interest.

## Publisher’s note

All claims expressed in this article are solely those of the authors and do not necessarily represent those of their affiliated organizations, or those of the publisher, the editors and the reviewers. Any product that may be evaluated in this article, or claim that may be made by its manufacturer, is not guaranteed or endorsed by the publisher.

## Footnotes

^1^https://www4.istat.it/it/archivio/207188

^2^https://magazine.wein.plus/news/three-consortia-of-lambrusco-producers-in-emilia-romagna-are-merging-new-protection-association-consorzio-tutela-lambrusco-monitors-eight-doc-areas

^3^https://www.consorziobalsamico.it/consortium/economic-data/?lang=en
