# Supplementary material for: Composition and biodiversity of soil and root-associated microbiome in Vitis vinifera cultivar Lambrusco distinguish the microbial terroir of the Lambrusco DOC protected designation of origin area on a local scale
Source: Front Microbiol. 2023 Feb 22;14:1108036. doi: 10.3389/fmicb.2023.1108036 (PMC9992870; doi:10.3389/fmicb.2023.1108036)
Supplement: Supplementary file 1 [file Data_Sheet_1.PDF]

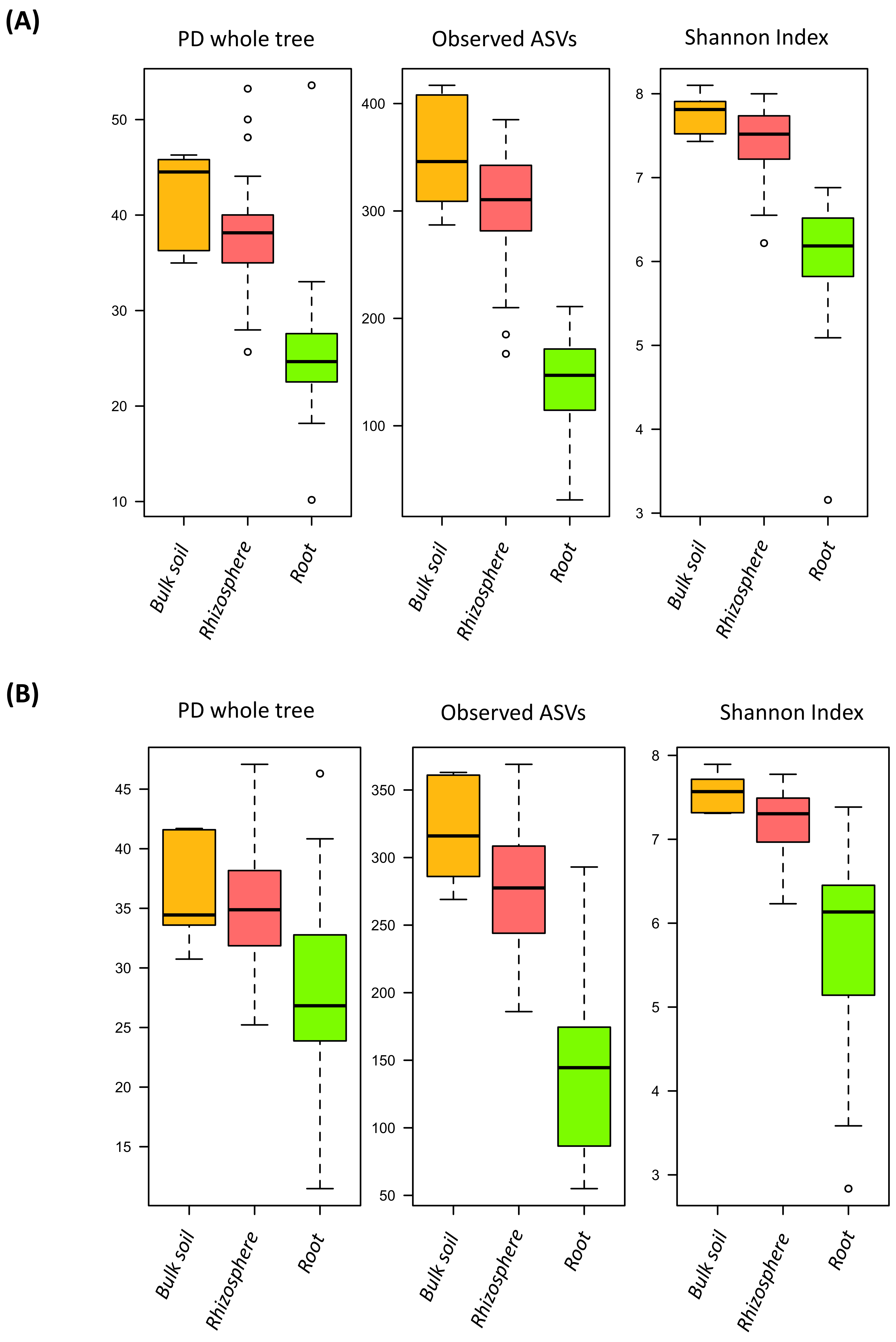

Supplementary Figure. S1 - Alpha diversity of bulk soil and of *V. vinifera* rhizosphere and root microbiomes in spring and fall. Box-plots showing the distributions of the Faith's Phylogenetic Diversity (PD whole tree), Observed ASVs and Shannon Index calculated for all samples of bulk soil and of *V. vinifera* (rhizosphere and root) in June **(A)** and November **(B)** 2021. According to all metrics, alpha diversity is higher in the bulk soil and in the rhizosphere and shows a steep decrease in the endophytic microbial communities ($P \leq 0.05$, Kruskall-Wallis test).

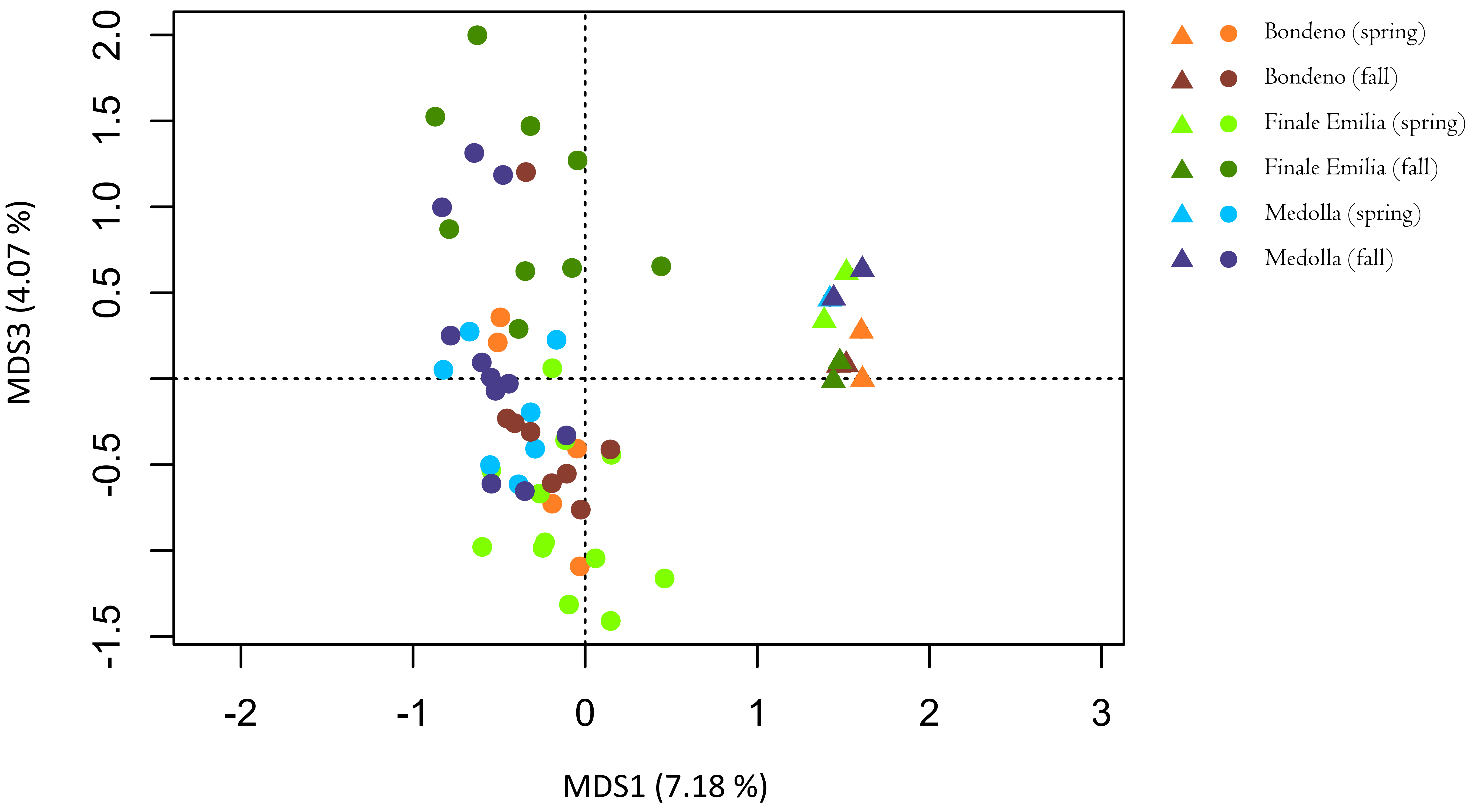

Supplementary Figure. S2 - Principal Coordinates Analysis (PCoA) based on unweighted UniFrac distances showing the variation of *V. vinifera* root (dots) and bulk soil (triangles) microbiomes across sites, i.e., Bondeno (orange-red), Finale Emilia (green) and Medolla (blue) and seasons (lighter shades for spring and darker shades for fall). The only significant differences emerge when comparing the two groups including all root samples and all bulk soil samples (permutation test with pseudo F-ratio, P ≤ 0.001). The first and third principal components (MDS1 and MDS3) are plotted and the percentage of variance in the dataset explained by each axis is highlighted.

(A)

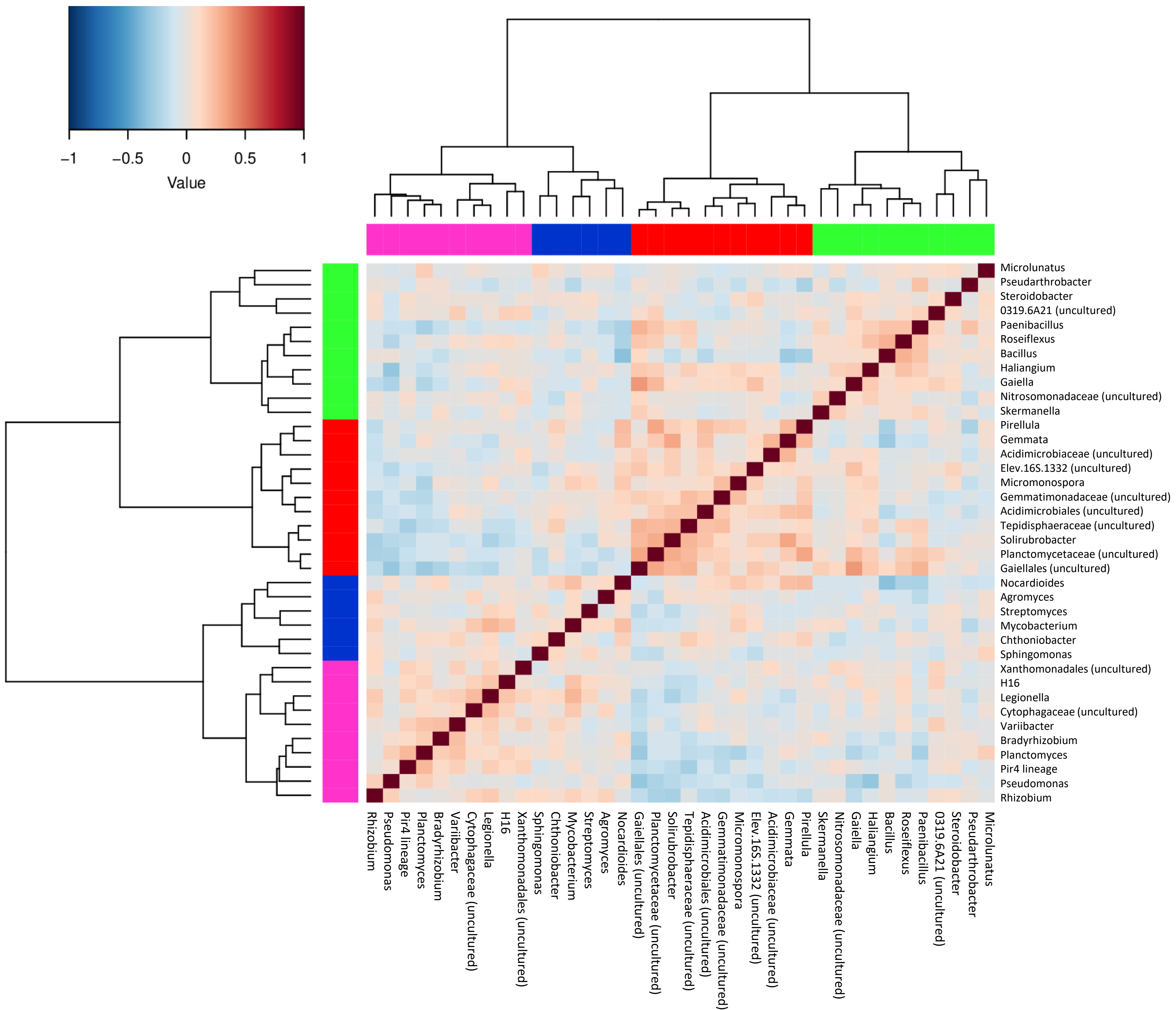

(B)

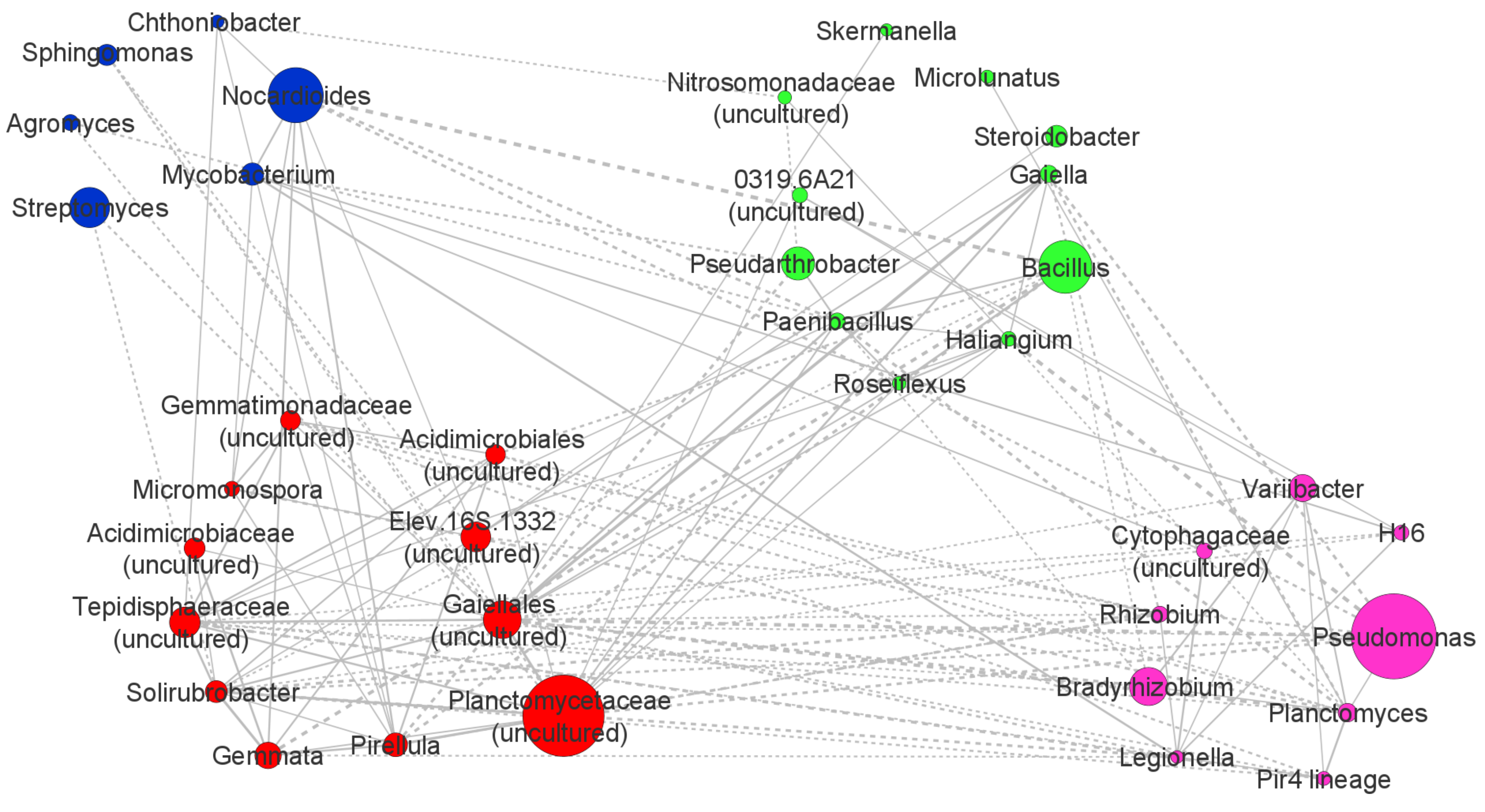

Supplementary Figure. S3 - Co-abundance associations between rhizospheric *V. vinifera* bacterial genera. **(A)** The assignment of co-abundance groups (CAGs) is based on a heat plot representing Kendall correlations between genera clustered by using the Spearman correlation coefficient and the Ward linkage hierarchical clustering method. Only genera whose relative abundance was higher than 0.5% in at least 33% of the samples are represented. Different colors indicate the four identified CAGs. **(B)** Wiggum plot correlations between the four identified CAGs. The size of the nodes is proportional to the mean genus abundance within the cohort and the connections between nodes represent positive (solid lines) and negative (dashed lines) significant Kendall correlations between genera (controlled for multiple testing using FDR, $P \leq 0.05$).
