# Supplementary material for: Composition and biodiversity of soil and root-associated microbiome in Vitis vinifera cultivar Lambrusco distinguish the microbial terroir of the Lambrusco DOC protected designation of origin area on a local scale
Source: Front Microbiol. 2023 Feb 22;14:1108036. doi: 10.3389/fmicb.2023.1108036 (PMC9992870; doi:10.3389/fmicb.2023.1108036)
Supplement: Supplementary file 2 [file Data_Sheet_2.PDF]

Supplementary Table S1. Sampling details of the present study

SITE	NUMBER OF SAMPLES COLLECTED				PDO AREA	AGRICULTURAL APPROACH
	**June 2021**		**November 2021**			
	**Plants**	**Soil**	**Plants**	**Soil**		
Bondeno	15	2	15	2	No	Traditional
Finale Emilia	15	2	15	2	Yes	Traditional
Medolla	15	2	15	2	Yes	Organic

Supplementary Table S2. Relative abundance in bulk soils of bacterial genera identified by LEfSe

	BACTERIAL GENERA	MEAN RELATIVE ABUNDANCE (%) (± SD)	
		**PDO bulk soil samples**	**Non-PDO bulk soil samples**
Enriched in PDO rhizospheres	*Bacillus*	1.24 (± 0.90)	0.66 (± 0.54)
	Pseudarthrobacter	0.39 (± 0.53)	0.44 (± 0.88)
	Planctomyces	0.41 (± 0.33)	0.37 (± 0.28)
	Gaiellales (uncultured)	4.03 (± 2.53)	2.75 (± 1.99)
	Skermanella	1.09 (± 0.97)	0.64 (± 0.48)
	Pir4 lineage	0.33 (± 0.23)	0.62 (± 0.34)
	Microlunatus	0.84 (± 0.88)	0.00 (± 0.00)
	Paenibacillus	0.63 (± 0.28)	0.42 (± 0.30)
Enriched in non-PDO rhizospheres	*Nocardioides*	1.86 (± 1.17)	1.56 (± 1.06)
	Micromonospora	0.70 (± 0.55)	1.01 (± 0.59)
	Gemmatimonadaceae (uncultured)	1.71 (± 0.71)	2.26 (± 0.97)
	Pirellula	1.14 (± 0.45)	1.47 (± 0.91)
	Legionella	0.16 (± 0.09)	0.04 (± 0.05)
	Mycobacterium	0.28 (± 0.33)	0.51 (± 0.57)
	Acidimicrobiales (uncultured)	1.15 (± 1.13)	1.49 (± 0.98)
	Chthoniobacter	0.45 (± 0.38)	0.26 (± 0.19)

SD (standard deviation)

Supplementary Table S3. PGP genes accession and version numbers (NCBI protein database)

Gene	Accession	Version
NifB	WP_011024080	WP_011024080.1
NifE	WP_014404757	WP_014404757.1
NifH	WP_010870393	WP_010870393.1
NifN	WP_011241560	WP_011241560.1
NifV	WP_011241567	WP_011241567.1
NifU	NP_461477	NP_461477.1
phoA	NP_414917	NP_414917.2
GDH	NP_388275	NP_388275.1
EntF	WP_000077784	WP_000077784.1
EntS	NP_415123	NP_415123.1
FslA	WP_003037766	WP_003037766.1
ipdC	WP_035671558	WP_035671558.1
aro10	NP_010668	NP_010668.3
aldH	NP_001260290	NP_001260290.1
AcdS	XP_037178185	XP_037178185.1

Supplementary Table S4. PGP genes K numbers (KEGG ORTHOLOGY database)

Gene	K number
NifB	K02585
NifE	K02587
NifH	K02588
NifN	K02592
NifV	K02594
NifU	K04488
phoA	K01077
GDH	K00034
EntF	K02364
EntS	K08225
FslA	-
ipdC	K04103
aro10	-
aldH	K00128
AcdS	K01505

Supplementary Table S5. PGP functions identified by PICRUSt2 (KEGG ORTHOLOGY database)

PGP function	Function-encoding bacteria	Function confirmed with PICRUSt2
Nitrogen fixation	*Bacillus*	No
	Unclassified Gaiellales	Yes
	Azospirillum/Skermanella	Yes
	Pirellulales	No
	Paenibacillus	Yes
	Nocardioides	No
Phosphorous solubilization	*Bacillus*	Yes
	Pseudarthrobacter	Yes
	Planctomyces	Yes
	Azospirillum/Skermanella	Yes
	Pirellulales	Yes
	Paenibacillus	Yes
	Nocardioides	Yes
	Micromonospora	Yes
	Mycolicibacterium	Yes
Siderophore production	*Bacillus*	No
	Pseudarthrobacter	Yes
	Azospirillum/Skermanella	No
	Pirellulales	No
	Microlunatus	Yes
	Paenibacillus	No
	Nocardioides	No
	Micromonospora	No
	Gemmatirosa	Yes
	Legionella	No
	Mycolicibacterium	Yes
	Unclassified Acidimicrobiales	No
	Chthoniobacter	No
IAA production	*Bacillus*	Yes
	Planctomyces	Yes
	Azospirillum/Skermanella	No
	Pirellulales	Yes
	Paenibacillus	Yes
ACC deaminase production	*Bacillus*	No
	Azospirillum/Skermanella	No
	Microlunatus	No
	Paenibacillus	No
	Nocardioides	Yes
	Unclassified Acidimicrobiales	No
